# Niclosamide is a potential therapeutic for familial adenomatosis polyposis by disrupting Axin-GSK3 interaction

**DOI:** 10.18632/oncotarget.16252

**Published:** 2017-03-16

**Authors:** Sung Yong Ahn, Nam Hee Kim, Kyungro Lee, Yong Hoon Cha, Ji Hye Yang, So Young Cha, Eunae Sandra Cho, Yoonmi Lee, Jeong Seok Cha, Hyun Soo Cho, Yoon Jeon, Young-Su Yuk, Suebean Cho, Kyoung Tai No, Hyun Sil Kim, Ho Lee, Jiwon Choi, Jong In Yook

**Affiliations:** ^1^ Department of Oral Pathology, Oral Cancer Research Institute, Yonsei University College of Dentistry, Seoul 03722, Korea; ^2^ Bioinformatics and Molecular Design Research Center, Yonsei University, Seoul 03722, Korea; ^3^ Department of Systems Biology and Division of Life Science, Yonsei University, Seoul 03722, Korea; ^4^ Graduate School of Cancer Science and Policy, Research Institute, National Cancer Center, Goyang 10408, Korea

**Keywords:** niclosamide, epithelial-mesenchymal transition (EMT), Axin-GSK3 interaction, Wnt signaling, familial adenomatosis polyposis (FAP)

## Abstract

The epithelial-mesenchymal transition (EMT) is implicated in tumorigenesis and cancer progression, and canonical Wnt signaling tightly controls Snail, a key transcriptional repressor of EMT. While the suppression of canonical Wnt signaling and EMT comprises an attractive therapeutic strategy, molecular targets for small molecules reverting Wnt and EMT have not been widely studied. Meanwhile, the anti-helminthic niclosamide has been identified as a potent inhibitor of many oncogenic signaling pathways although its molecular targets have not yet been clearly identified. In this study, we show that niclosamide directly targets Axin-GSK3 interaction, at least in part, resulting in suppression of Wnt/Snail-mediated EMT. *In vitro* and *in vivo*, disruption of Axin-GSK3 complex by niclosamide induces mesenchymal to epithelial reversion at nM concentrations, accompanied with suppression of the tumorigenic potential of colon cancer. Niclosamide treatment successfully attenuates Snail abundance while increasing E-cadherin abundance in xenograft tumor. Notably, oral administration of niclosamide significantly suppressed adenoma formation in an APC-MIN mice model, indicating that niclosamide is an effective therapeutic for familial adenomatosis polyposis (FAP) patients. In this study, we identified a novel target to control the canonical Wnt pathway and Snail-mediated EMT program, and discovered a repositioned therapeutics for FAP patients.

## INTRODUCTION

As the EMT comprises a biological mechanism that induces invasive and stemness properties with therapeutic resistance in human cancer [1], reverting EMT with small molecule inhibitors is an attractive therapeutic strategy for cancer [2]. It has been found that salinomycin can effectively revert EMT, resulting in significant suppression of cancer stemness by 100-fold relative to paclitaxel and of metastatic potential of breast cancer cells *in vivo* [2]. Although salinomycin is limited for human, this finding suggests a novel strategy for regulating EMT in human cancer.

The transcription factor Snail triggers EMT in human cancer by suppressing epithelial genes [3]. Previous studies reveal that major oncogenic pathways, such as canonical Wnt signaling, p53 tumor suppressor, and bacterial carcinogen CagA protein of H. *pyroli*, modulate Snail activities via post-transcriptional and post-translational mechanisms [4–6]. Importantly, β-catenin and Snail transcriptional machinery are phosphorylated and degraded by GSK3, and canonical Wnt signaling or CagA inhibits the phosphorylation, consequently increasing TCF transcriptional activity and driving the Snail-mediated EMT program [4–8]. Axin2, a GSK3 scaffolding protein, plays a key regulatory function in this process by regulating nuclear-cytoplasmic shuttling of GSK3, resulting in increased nuclear Snail in cancer cells [8, 9]. The GSK3 shuttling function by Axin is also required for phosphorylation of the membranous LRP6 Wnt co-receptor and subsequent activation of intracellular Wnt signaling [10]. These observations indicate that Axin-GSK3 complex may play an important role in regulating canonical Wnt signaling and the Snail-mediated EMT program. Conversely, targeted disruption of the Axin-GSK3 complex can be a novel mode of action (MoA) in developing a small molecule inhibitor targeting Wnt signaling and the Snail-mediated EMT program for human cancer.

Niclosamide is an FDA-approved anti-helminthic drug used worldwide against intestinal cestodes infection for nearly 50 years [11]. Since niclosamide was reported as an effective agent for human colon cancer *in vitro* and *in vivo* [12, 13], a large body of recent studies have revealed that it can be used for various types of human cancers [14, 15]. Based on these observations, we addressed two concerns regarding the MoA of niclosamide. First, niclosamide induced cancer cell death at μM concentration level *in vitro* while physiological concentration *in vivo* exhibited nM level in plasma and cancer tissue [12, 13], indicating a non-cytotoxic MoA *in vivo*. Second, while many targets, such as Notch signaling, Dishevelled, S100A4, and Frizzled receptor, have been proposed [12–14], the direct target providing the MoA of the niclosamide has not yet been clearly identified.

In this study, we found that niclosamide directly disrupts the Axin-GSK3 complex, at least in part, resulting in attenuation of canonical Wnt activity with reversion of Snail-mediated EMT in colon cancer cells. Further, niclosamide attenuates TCF/LEF transcriptional activity induced by APC mutants, and oral administration of niclosamide significantly suppresses adenoma formation in an APC-MIN model, of clinical relevance for familial adenomatosis polyposis (FAP) patients. Considering the limited treatment options for and clinical complications of FAP, our observations suggest a potential therapeutic for FAP patients based on a novel target and MoA.

## RESULTS

### Niclosamide attenuates canonical Wnt activity with reversal of Snail-mediated EMT at nM levels in colon cancer cells

**Figure 1:**
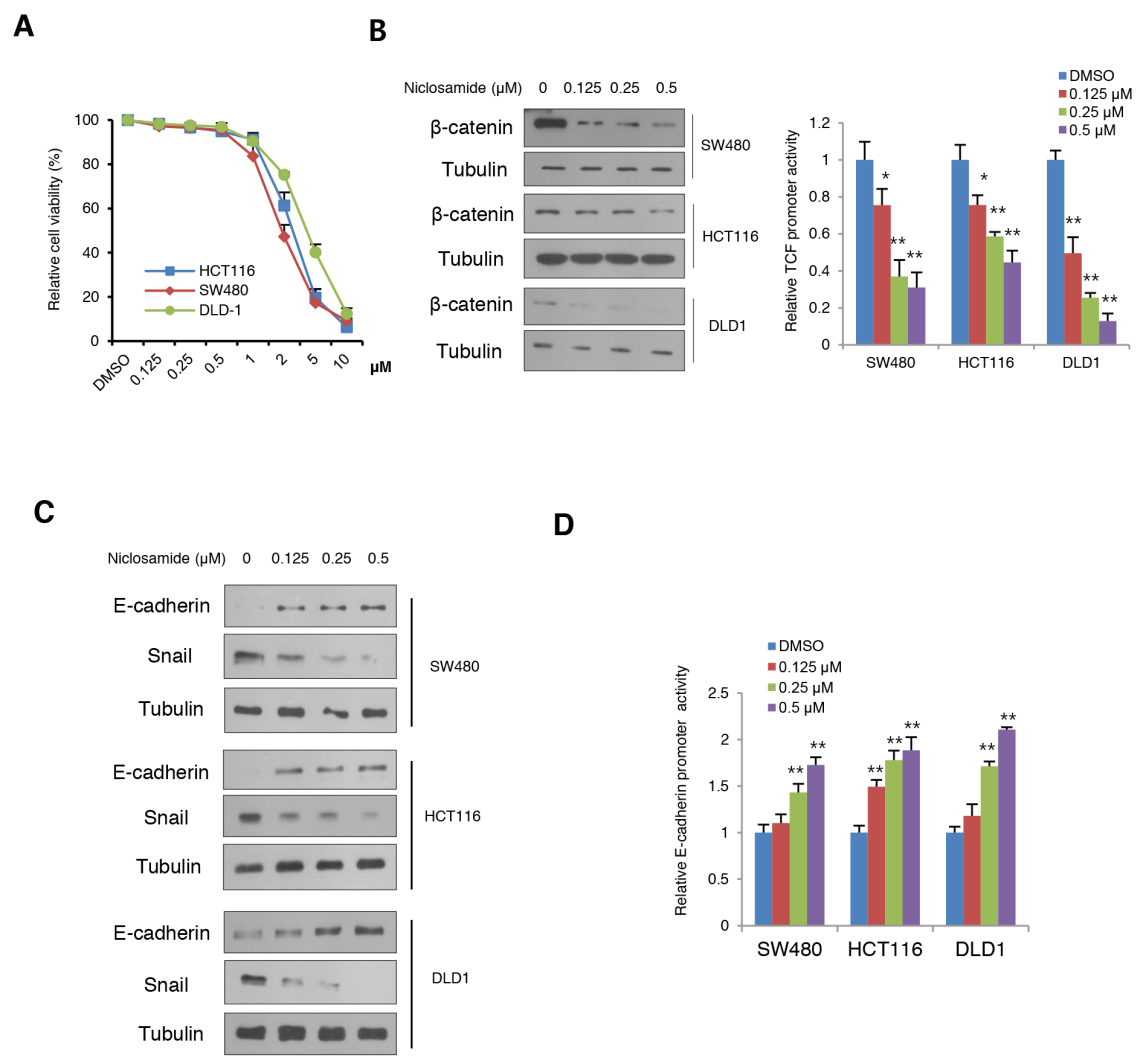
Niclosamide reverses Snail-mediated EMT at nM levels with suppression of canonical Wnt signaling **(A)** Cell viability of colon cancer cells after treatment of niclosamide for a 48-h period. Cell viability was determined by trypan blue exclusion assay from triplicate experiments. **(B)** Effect of niclosamide on β-catenin abundance (left panels) and TCF/LEF reporter (Topflash) activity (right panel) in colon cancer cells transiently transfected with Topflash with *Renilla* luciferase and treated with different concentrations of niclosamide (0, 0.125, 0.25, and 0.5 μM) for 24 h. The reporter activity was determined by measuring the luciferase normalized with *Renilla* from triplicate experiments. **(C)** Niclosamide suppresses Snail-mediated EMT. The colon cancer cells were treated with niclosamide at the indicated concentrations for 24 h, and protein abundance of Snail and E-cadherin was determined by immunoblot analysis. **(D)** Colon cancer cells were transiently transfected with wild type or 3x mutant E-cadherin proximal reporter vector with *Renilla* control. Cells were treated with different concentrations of niclosamide for 24 h. The reporter activity was determined by measuring the luciferase activity normalized with *Renilla* activity from triplicate experiments. Relative reporter activity of wild type E-box compared to 3x E-box mutant reporter is presented. Statistical significances compared to control are denoted as *, P < 0.05; **, P < 0.01 by a two-tailed Student's t-test.

While earlier studies focused on cell death at μM levels *in vitro*, we hypothesized that niclosamide inhibits the EMT process in a physiologically relevant nM concentration [12, 13]. To examine this hypothesis, we first screened cancer cell death with variable concentration in colon cancer cells and found that niclosamide induced cell death at μM level whereas nM concentrations did not (Figure 1A). We then evaluated β-catenin protein abundance and TCF/LEF transcriptional activity with reference to physiological levels of niclosamide in colon cancer cells. Consistent with previous observations [12], niclosamide suppressed β-catenin protein abundance and TCF/LEF activity in a dose-dependent manner (Figure 1B). Because the canonical Wnt pathway directly up-regulates the Snail-mediated EMT program of cancer cells [7, 8], we next examined Snail and E-cadherin protein abundance. Examining protein abundance of Snail and E-cadherin, we found niclosamide treatment suppressed Snail abundance while increasing E-cadherin in colon cancer cells (Figure 1C). Given the well-known function of Snail as an E-cadherin transcriptional repressor [16, 17], nM levels of niclosamide were sufficient to increase E-cadherin promoter activities (Figure 1D).

**Figure 2:**
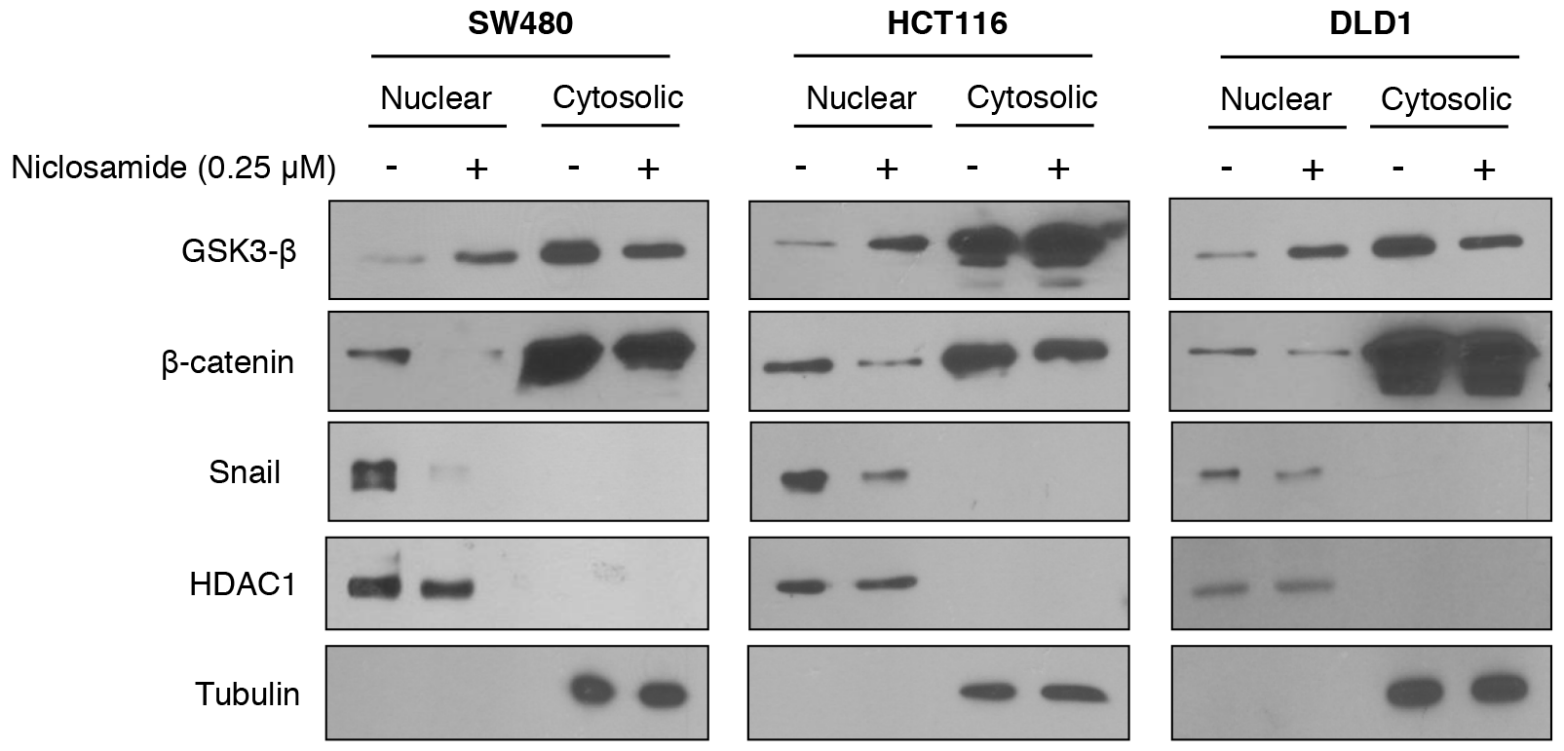
Niclosamide increased nuclear GSK3 activity resulting in decreased nuclear β-catenin and Snail abundance The colon cancer cells were treated with 0.25 μM niclosamide for 24 h, and the protein abundance of β-catenin and Snail in nuclear-cytosolic fraction was determined by immunoblot analysis. HDAC1 and tubulin served as the loading control for nuclear and cytosolic fractions, respectively.

As a scaffolding protein of the Wnt pathway [18, 19], Axin2 shuttles GSK3 to increase membranous LRP6 phosphorylation/stabilization and to decrease nuclear GSK3 activity, activating intracellular signaling of Wnt and Snail-mediated EMT [8, 10]. Although earlier studies have suggested that Axin is a tumor suppressor, recent evidence supports the important role of Axin2 in canonical TCF/LEF activity as well as in Snail-mediated EMT progression [8, 9, 20]. As a TCF/LEF target gene, the Axin2 is highly expressed in colon cancer due to loss of APC tumor suppressor function or β-catenin mutation [18, 19, 21], and we validated increased Axin2 abundance in colon cancer cell panels (Supplementary Figure 1A). Next, we examined whether Axin2 regulates TCF/LEF transcriptional activity and the Snail-mediated EMT program in colon cancer cells. Consistent with previous observations [9], inducible knock-down of Axin2 exhibited suppression of canonical Wnt activity in tandem with reversion of Snail-mediated EMT, similarly to niclosamide treatment (Supplementary Figure 1B-1E). Because Axin2 is required for nuclear and membranous GSK-3 dynamics [8–10], we next examined abundance of nuclear GSK3 and phosphorylated LRP6. Indeed, niclosamide treatment increased nuclear GSK3 whereas β-catenin and Snail abundances were suppressed in a colon cancer panel (Figure 2). The phosphorylation and protein abundance of LPR6, a Wnt co-receptor, were also significantly suppressed by niclosamide (Supplementary Figure 2). These data support that niclosamide may affect Axin function in colon cancer cells.

### Niclosamide directly binds to GSK3, disrupting the Axin-GSK3 complex

**Figure 3:**
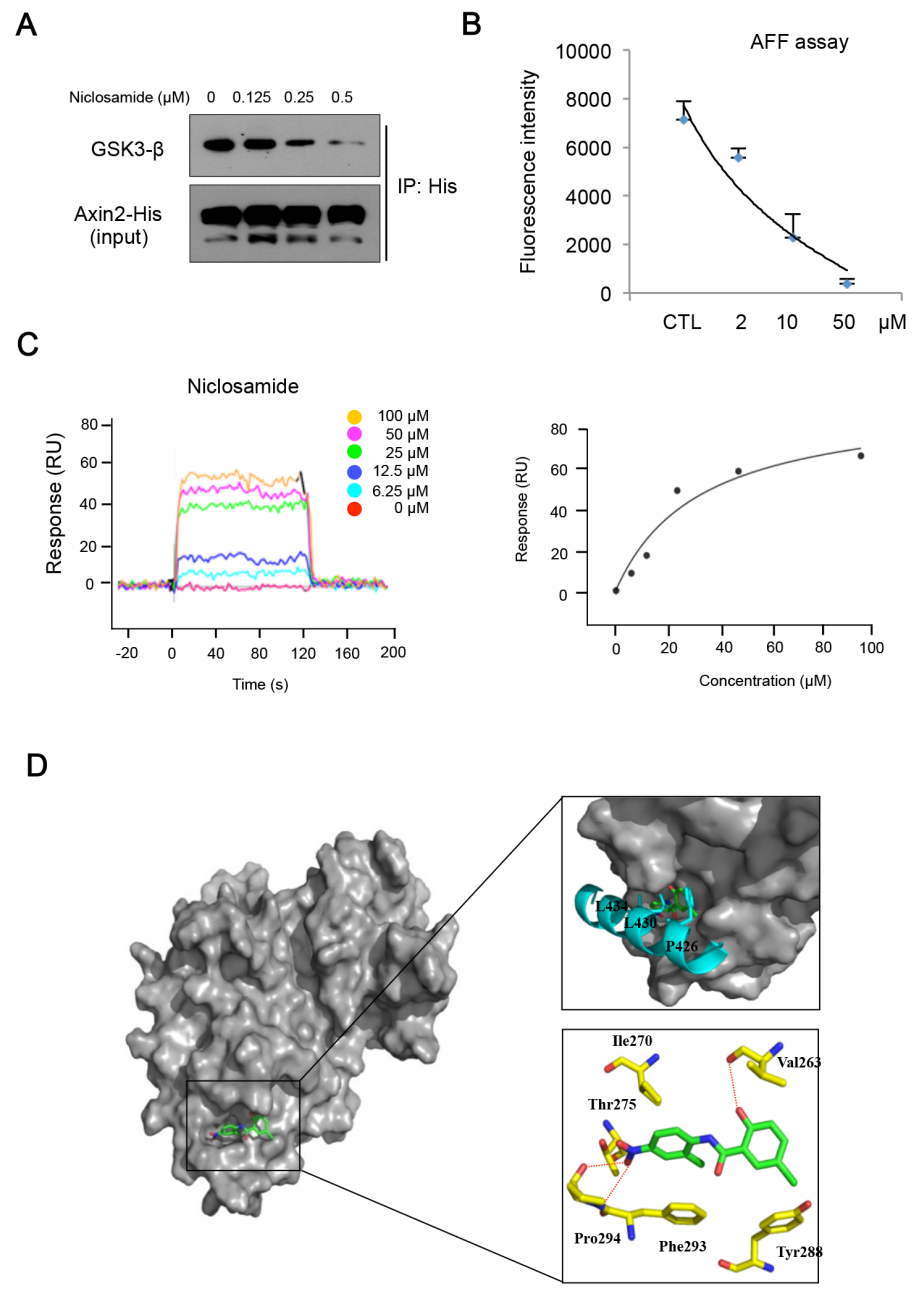
Niclosamide directly interacts with GSK3, inhibiting Axin binding **(A)** The 293 cells were transfected with His-tagged Axin2 expression vector and treated with increasing doses of niclosamide for an 8 h period. The GSK3 binding activities in lysate were determined by Ni-NTA bead immunoprecipitation followed by immunoblot analysis for GSK3. **(B)** Recombinant His-tagged GSK3 was subjected to immunoprecipitation to determine FITC-conjugated Axin peptide (19 mer) binding. Remaining fluorescent intensities following incubation with different doses of niclosamide are presented from triplicate experiments. **(C)** Surface plasmon resonance (SPR) sensograms showing the interaction of niclosamide with immobilized GSK3 (left panel). Five different niclosamide concentrations were analyzed (0 - 100μM). Response units at equilibrium are plotted against the concentration of niclosamide (right panel). The data were analyzed using the ProteOn Manager Software 2.0. **(D)** Predicted binding mode of niclosamide on the Axin-binding site of GSK3 shown as surface model (left panel). Superimposition of the Axin peptide and niclosamide to demonstrate their similar binding modes within the peptide binding site of GSK3 (top right panel). Detailed interactions between niclosamide and GSK3 are shown as stick model H-bonds are indicated by red dashed lines (bottom right panel). GSK3, Axin peptide, and niclosamide are colored in yellow, cyan, and green, respectively. Figures were drawn using PyMol (Delano Scientific LLC, San Carlos, CA).

During the Wnt signal transduction, Axin serves as a scaffolding protein that directly interacts with GSK3 [22], and Axin1 and Axin2 proteins are functionally equivalent *in vivo* [23]. We validated Axin2 functions as equivalent to Axin1 in terms of GSK3 binding and nuclear export function based on structural analysis (Supplementary Figure 3A) [24]. To determine whether niclosamide can inhibit Axin-GSK3 interaction, we subjected the lysate of cells expressing full-length Axin2 to immunoprecipitation in absence or presence of niclosamide. Indeed, niclosamide decreased GSK3 binding to Axin2 in cells and *in vitro* cell lysate samples (Figure 3A & Supplementary Figure 3B). Previous structural analysis of Axin-GSK3 binding indicated that hydrophobic residues on α helix of Axin are packed into a hydrophobic groove formed by C-terminal loops in GSK3 [24]. Thus, we hypothesized that niclosamide can bind to the hydrophobic groove on GSK3, inhibiting Axin function. To test this notion, we next designed a cell-free assay system for competitive inhibition by niclosamide of recombinant GSK3 binding to 19-mer FITC-conjugated Axin peptide (AFF assay). Interestingly, niclosamide disrupted interaction between recombinant GSK3 and synthetic Axin peptide in a dose-dependent manner in AFF assay (Figure 3B), indicating that niclosamide inhibits Axin-GSK3 binding in a cell-free system. We next performed surface plasma resonance (SPR) analysis to determine whether there was direct binding of niclosamide to GSK3. As shown in the sensograms (Figure 3C and Supplementary Figure 3C), wild type Axin peptide or niclosamide directly binds to GSK3, the equilibrium dissociation constant yielding a K_D_ valueof 11.9 μM and 34.5 μM in SPR analysis, respectively. Mutant Axin peptide having mutations on hydrophobic residues at GSK3 protein immobilized on the sensor surface did not bind up to 20μM. In general, a high-affinity interaction is characterized by a low K_D_ value, the rapid recognition and binding of the interactants (high *k_a_* value), and the stability of the complex formation (low *k_d_* value). The niclosamide had lower *k_a_* and *k_d_* values comparable to that of wild type Axin peptide, indicating that niclosamide has efficient recognition and a slower rate of dissociation, and thus form stable complexes with GSK3 (Table 1). This SPR analysis result was consistent with the observation from AFF assay that niclosamide disrupts Axin-GSK3 interaction and blocks Axin function by competitive binding to GSK3. To further investigate the interaction of niclosamide on the Axin-binding site of GSK3 structurally, a molecular docking study was performed. The 1-chloro-3-nitrobezene group of niclosamide docks into a hydrophobic cavity formed by residues Val 263, Leu 266, Val 267, and Ile 270 of human GSK3β and is stacked onto residue Phe 293 through π–π interactions (Figure 3D). Niclosamide additionally forms hydrogen bonds with Pro294, Thr275, and Val 263, and halogen bonds with Tyr288 on the Axin-GSK3 interaction surface [24, 25]. These results indicate that niclosamide disrupts the Axin-GSK3 complex by inhibiting protein-protein interactions (PPI).

**Table 1:** Affinity and kinetic data for GSK3 interactions determined by SPR analysis

Compound	*k_a_*, M^−1^sec^−1^	*k_d_*, sec^−1^	*K_D_*_, μ_M
Niclosamide	1.24E+04	4.26E-01	3.45E-05
Axin peptide	8.25E+04	9.83E+01	1.19E-05

The association rate (*k_a_*), dissociation rate (*k_d_*) and equilibrium dissociation constant (*K_D_*) of each virtual hit are shown in the table. *K_D_* values were calculated by the individual *k_a_* and *k_d_* values.

**Figure 4:**
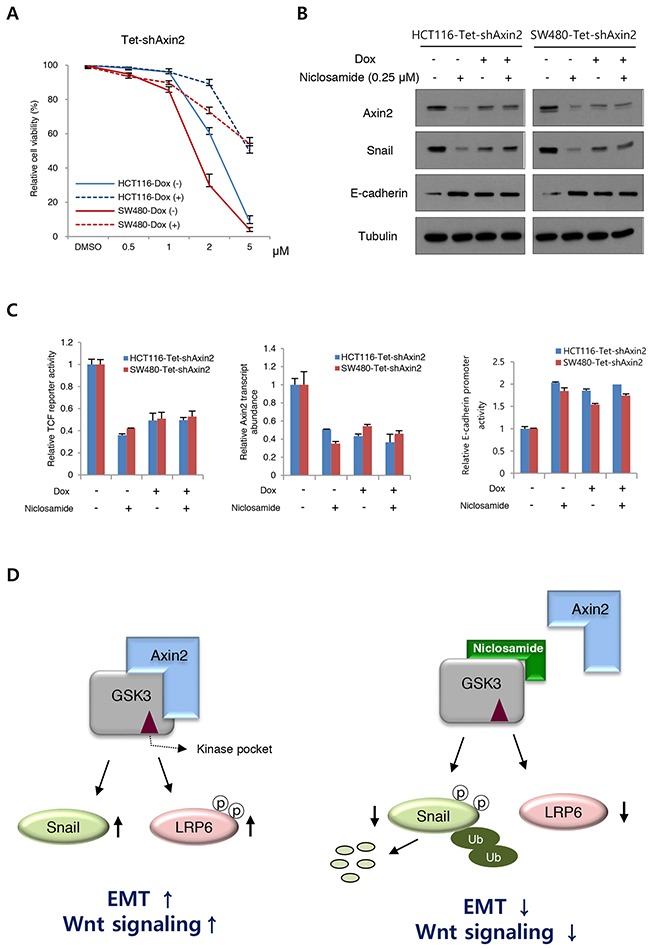
On-target effects of niclosamide in colon cancer cells **(A)** Cell viability of colon cancer cells after niclosamide treatment were measured following inducible knockdown of Axin2 (Dox +). **(B)** Niclosamide regulates EMT genes in an Axin2-dependent manner. The colon cancer cells expressing inducible shRNA for Axin2 (Dox+) were treated with niclosamide (0.25 μM), and indicated protein abundance was determined by immunoblot analysis. **(C)** The TCF/LEF reporter activity (left panel), Axin2 transcript abundance (middle panel), and E-cadherin reporter activity (right panel) in colon cancer cells expressing inducible knockdown of Axin2 were determined under niclosamide treatment (0.25 μM). **(D)** Schematic diagram of mechanism of action of niclosamide. In the absence of niclosamide, the Axin2 binds to GSK-3, resulting in up-regulation of Snail-mediated EMT and canonical Wnt activity (left panel). Disruption of Axin-GSK3 interaction by niclosamide allows degradation of Snail and attenuation of canonical Wnt activity (right panel).

To determine on-target effects of niclosamide on cells, we next made Axin2 knockdown cells using a doxycycline-inducible system. Evaluating the cytotoxic effects of niclosamide, we found the cell viability was largely increased at μM levels by inducible Axin2 knockdown in HCT116 and SW480 cells (Figure 4A). Examining protein abundance of Snail and E-cadherin, the effects of niclosamide were largely abolished by Axin2 knockdown (Figure 4B). Further, canonical Wnt transcriptional activity, Axin2 transcript abundance, and E-cadherin reporter activity were minimally changed by niclosamide treatment in Axin2 knockdown colon cancer cells (Figure 4C), indicating that Axin2 is required for mode of action (MoA) of niclosamide on colon cancer cells. Therefore, niclosamide suppresses canonical Wnt activity and the Snail-mediated EMT program in an on-target manner by inhibiting Axin-GSK3 interaction (Figure 4D).

Previously, we have reported that *Helicobacter pylori* CagA binds to GSK3, resulting in depletion of GSK3 activity similarly to Axin and subsequent potentiation of Snail-mediated EMT [6]. To determine whether niclosamide can inhibit CagA binding to GSK3, we then transfected CagA expression vector and performed immunoprecipitation and immunoblot assay with niclosamide. Intriguingly, niclosamide also inhibited CagA-GSK3 interactions in cell lysates (Supplementary Figure 3D), and niclosamide treatment attenuated Snail protein abundance that is stabilized by CagA in a dose-dependent manner (Supplementary Figure 3E). These data further support that niclosamide inhibits binding of *H*. CagA as well as of Axin onto GSK3, resulting in reversion of Snail-mediated EMT.

### Niclosamide reverts the Snail-mediated EMT program *in vivo*

**Figure 5:**
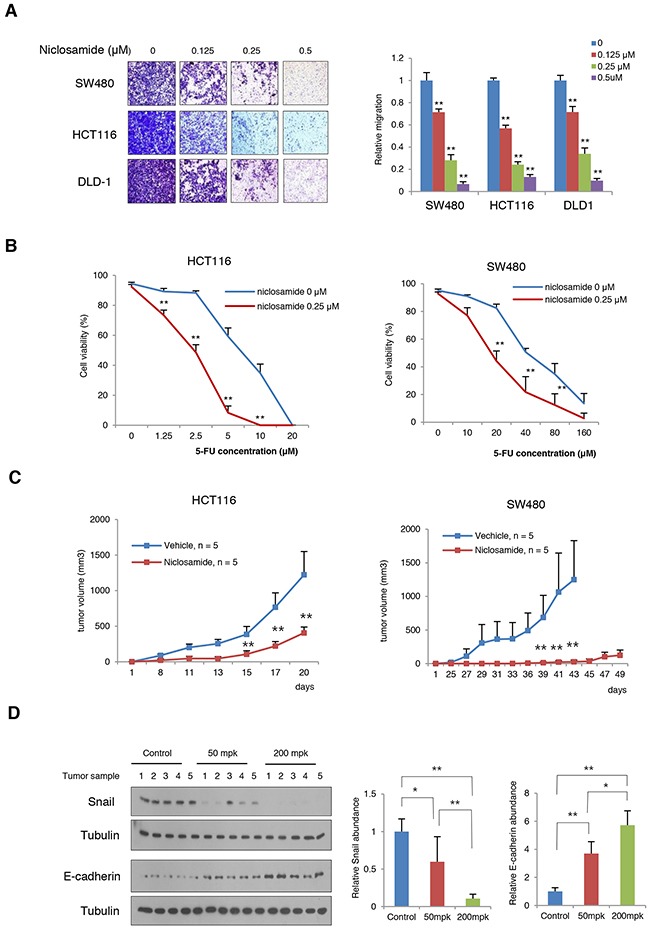
Niclosamide suppresses tumorigenic potential and EMT *in vivo* **(A)** The migratory activity of colon cancer cells treated with various concentrations of niclosamide was determined by transwell migration assay (left panels). Statistical significances compared to control (right panel) are denoted as **, P < 0.01 by a two-tailed Student's t-test. **(B)** Combination effect of niclosamide and 5-FU on HCT116 and SW480 cells. The colon cancer cells were treated with various concentrations of 5-FU and niclosamide as indicated. Cell viability was measured trypan blue exclusion assay after 48 h of incubation. **, P < 0.01 by a two-tailed Student's t-test. **(C)** Niclosamide inhibits the growth of colon cancer cells *in vivo*. HCT116 and SW480 colon cancer cells were inoculated into the flank of athymic nude mice prior to 24 h of treatment. Niclosamide (50 mg/kg dissolved in Cremophor EL) was intraperitoneally administered. Two asterisks, P < 0.01 compared to the vehicle control by Mann-Whitney test. **(D)** Niclosamide treatment reversed Snail-mediated EMT in SW480 xenografts. Tumor-bearing nude mice were given 50 mg/kg or 200 mg/kg body weight niclosamide for 3 days prior to sacrifice. Tumor tissues were collected and the protein abundances of Snail and E-cadherin in tumor lysates were then measured using immunoblot assay (left panels). The band intensity on each blot was normalized to the loading control and the relative protein abundance is shown as the ratio to that in the vehicle control (middle and right panels). Results are shown as means and s.d. One asterisk, P < 0.01; Two asterisks, P < 0.001 compared to the vehicle control by Mann-Whitney test.

The Wnt activation and Snail-mediated EMT promote cell migration and tumorigenic potential along with increased therapeutic resistance. When we treated the niclosamide at nM level, the migration potential of colon cancer cells was significantly inhibited (Figure 5A). Conversely, when Axin and Snail levels were increased with tankyrase inhibitor XAV939, cell migratory potential significantly increased (Supplementary Figure 4) [9, 26], supporting that Axin2 serves tumor progression in colon cancer cells. Further, niclosamide treatment sensitized cytotoxic effect of 5-fluorouracil (5-FU) in colon cancer cells compared to control (Figure 5B). Next, we examined the effect of niclosamide on *in vivo* tumorigenic potentials in HCT116 and SW480 cells. Consistent with previous observations [12, 13], intraperitoneal administration of niclosamide significantly suppressed *in vivo* tumor growth of colon cancer cells (Figure 5C). For further evaluation of the *in vivo* MoA of the niclosamide suppressing Snail-mediated EMT, tumor xenograft tissues were collected 3 days after treatment of vehicle or niclosamide (50 and 200 mg per kg, body weight), and the protein abundance of Snail and E-cadherin in tumor extracts were analyzed. Indeed, Snail abundance in *in vivo* samples was decreased whereas E-cadherin abundance was increased by niclosamide treatment in a dose-dependent manner (Figure 5D). These results indicate that niclosamide suppresses tumorigenic potential *in vivo* by reverting Snail-mediated EMT.

### Oral administration of niclosamide suppresses adenoma formation in APC-MIN mice

**Figure 6:**
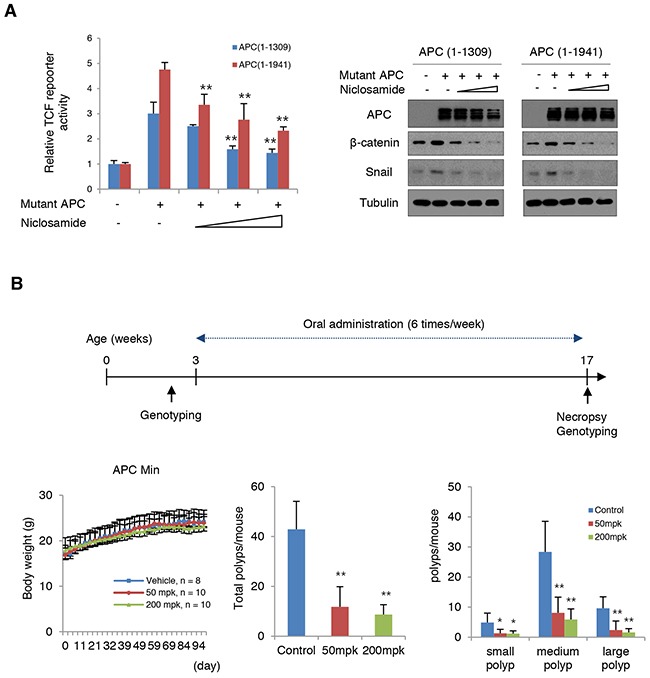
Niclosamide attenuates Wnt activity induced by mutant APC **(A)** Niclosamide suppressed TCF/LEF transcriptional activities induced by mutant APC in a dose-dependent manner. The 293 cells were transiently transfected with mutant APC (1-1309 or 1-1941) and Topflash reporter vector. Cells were treated with increasing concentrations of niclosamide (0, 0.125, 0.25, and 0.5 μM) for 24 h. TCF/LEF transcriptional activities were quantified by measuring relative luciferase activity normalized to *Renilla* (left panel). The lysates of reporter assay were subjected to immunoblot assay for β-catenin and Snail (right panels). **(B)** Schematic diagram of experimental design for oral administration into APC-Min mice model (upper). Body weight (left), total number of polyps (middle), and size distribution of polyps (right) in small intestine (small, < 1 mm; medium 1~3 mm; large > 3 mm). Results are shown as means and s.d. One asterisk, P < 0.01; Two asterisks, P < 0.001 compared to the vehicle control by Mann-Whitney test.

The adenomatous polyposis coli (APC), most commonly mutated in sporadic colon cancer, is a key tumor suppressor gene that acts as a gatekeeper of intestinal epithelial homeostasis by restricting cytosolic β-catenin [27–30]. Mutational inactivation of APC is also a well-known genetic background of FAP patients. Axin2, a representative downstream target of TCF/LEF transcriptional machinery, is highly abundant in colon cancer cells as well as in adenomas due to loss of APC function [18, 19, 21]. Given the observations that niclosamide suppresses canonical Wnt activity and EMT via Axin-GSK3 inhibition, we next tested whether niclosamide could attenuate TCF/LEF transcriptional activity and Snail abundance induced by mutant APC. When we transfected mutant APC in 293 cells, TCF/LEF transcriptional activity increased and niclosamide treatment relieved this activity together with decreased ß-catenin and Snail abundance in a dose-dependent manner (Figure 6A), indicating that niclosamide effectively attenuates canonical Wnt activity and Snail abundance induced by APC mutation. To prove functional relevance *in vivo*, we preliminarily tested the effect of niclosamide on adenoma formation in APC-MIN (multiple intestinal neoplasia, APC^Δ850^) mice model. APC-MIN mice (3 weeks old) were intraperitoneally injected with daily doses of vehicle as control (n = 6, 6 times/week) or 50 mg/kg niclosamide (n = 7, 6 times/week). Fourteen weeks after niclosamide administration, the intestinal adenoma burden was significantly decreased while the body weight was unaffected (Supplementary Figure 5A). FAP having an inherited APC germ-line mutation results in multiple adenomatous polyps and a nearly 100% risk of colon cancer before age 40 [31, 32]. While several adjunctive therapies with COX or ornithine decarboxylase inhibitors have been approved in patients with FAP, long-term follow up results were limited on adenoma formation, with increased risk of various complications, such as cardiovascular events [33, 34]. Upregulation of Axin2 and Snail abundance in APC-MIN adenoma or precancerous sporadic adenoma has been well-reported [18, 35], and Snail inhibition using anti-sense morpholino suppressed adenoma formation in APC-MIN model [36]. To examine the clinical relevance of our observations, we next designed *in vivo* experiments to test the therapeutic availability of niclosamide for FAP patients. APC-MIN mice were orally administrated 6 times/week with daily doses of vehicle (15% sugar gel) as control (n = 8) or 50 mg/kg niclosamide (n = 10) or 200 mg/kg niclosamide (n = 10). Interestingly, oral administration of niclosamide for 14 weeks end-point significantly suppresses intestinal adenoma formation in APC-MIN model, with no apparent dose-dependency (Figure 6B and Supplementary Figure 5B). The mice were stable during the drug treatment. These results suggest the prospect of niclosamide as a repositioned drug for FAP patients.

## DISCUSSION

Niclosamide (trade name Niclocide in US) is an orally available drug having antihelmintic and potentially antineoplastic activity. Due to its effectiveness and low cost, WHO has designated niclosamide as an essential medication in a basic health plan. Although its molecular target was not clearly identified, recent evidence suggests niclosamide may function as an anticancer drug by regulating many signaling pathways, such as Wnt, S100A4, Notch, and androgen receptor [12–14]. In this study, we provide evidence that niclosamide inhibits Axin binding to GSK3, resulting in attenuation of canonical Wnt activity and reversion of the Snail-mediated EMT program in colon cancer cells. We also provide clinical relevance of niclosamide as a potential therapeutics for FAP patients.

Recent observation suggests that many canonical Wnt target genes play an important role in EMT of colon cancer and are involved in crosstalk with metastatic potential [37]. Thus, understanding the key regulatory networks and functional mediators of the EMT program provides the opportunity to develop a novel therapeutic strategy. In this study, we focused on regulation of Snail-mediated EMT with small molecule niclosamide, which disrupts the Axin-GSK3 complex involved in canonical Wnt signaling. Our observations provide several functional and clinical insights relevant to cancer therapeutics involving reversion of the Snail-mediated EMT program.

First, we identified GSK3-Axin2 interaction as a molecular target of niclosamide, providing a novel MoA to suppress canonical Wnt activity through reversion of the Snail-mediated EMT program in cancer cells. Although it is hard to prove the existence of an off-target effect, our *in vitro* and *in vivo* evidence support that niclosamide exerts a biological effect by disrupting the Axin-GSK3 complex, at least in part. Secondly, aberrant activation of the Wnt pathway is associated with a variety of diseases, including cancer, bone metabolism, degenerative diseases, and fibrosis [38]. Despite the many proposed Wnt therapeutic targets, such as Frizzled receptor, Porcupine, Dishevelled, p300, and CBP (CREB-binding protein), effective molecular targets for regulating the canonical Wnt pathway remain limited [38]. During the Wnt activation, the APC-Axin-Dishevelled scaffolding complex is a key regulator of TCF/LEF activity. We found Axin2-GSK3 interaction to be a novel target for attenuating canonical Wnt activity and subsequent reversion of the Snail-mediated EMT program. Based on the structure of the protein-protein interaction (PPI) [24, 39], development of a new small molecule that specifically inhibits Axin-GSK3 PPI holds potential as a new cancer therapeutic. Third, we have previously shown that the Snail-mediated EMT program is tightly controlled by the canonical Wnt pathway and the p53 tumor suppressor [4, 5]. Based on experimental evidence with salinomycin [2], several EMT targets (mainly involving TGF-β signaling) for pharmacologic agents have been proposed [40]. In this study, we provide experimental evidence that the Snail-mediated EMT program closely coupled with the canonical Wnt pathway constitutes a promising nexus for human cancer therapeutics. Lastly, germ-line defects in APC cause FAP, in which affected patients develop hundreds of adenomatous polyps at an early age and ultimately progress to colorectal adenocarcinoma with 100% penetrance [31, 32]. While the US FDA and European Medicines Agency approved several anti-inflammatory drugs as adjunct treatments [32], targeted therapeutics for the Wnt pathway are still limited. We showed that orally administered niclosamide can be effectively repositioned drug for FAP. While a dose-dependent regression of adenomas was not found, probably due to the daily administration design, the efficacy in APC-MIN mice reported in the present study strongly supports the clinical relevance of niclosamide for FAP. Further studies of dosage and long-term toxicity are needed to confirm its therapeutic potential and clinical benefit for FAP patients.

## MATERIALS AND METHODS

### Cell lines and reagents

Colon cancer cell lines (HCT116, SW480 and DLD1) and 293 cells from ATCC were maintained under conditions recommended by the provider. Niclosamide (Cayman), tankyrase inhibitor XAV939 (X3004, Sigma), and 5-flulorouracil (Sigma) were solubilized in DMSO for *in vitro* experiments. E-cadherin reporter gene construct and Topflash reporter having 8x TCF/LEF binding sites were used as described previously [4, 7, 8]. The Tet-pLKO-puro vector (#21915 obtained from Addgene) was used for inducible shRNA knockdown. The target sequence of shRNA for Axin2 was 5′-accaccactacatccacca. Flag-tagged *Helicobacter pyroli* CagA, HA-tagged GSK3β, His-tagged Axin2, and Snail expression vector were described previously [6]. Mutant APC expression vectors pCMV-neo-Bam APC 1-1309 (#16508) and pCMV-neo-Bam APC 1-1941 (#16510) were obtained from Addgene.

### Cell viability, cell migration

For cell viability assay, 1 × 10^5^ cells were plated into 6-well plates with normal culture medium one day before niclosamide treatment. The cells were washed with PBS and cultured in culture medium for 48 h with niclosamide or in combination with 5-FU. Cell death was measured by trypan blue exclusion assay and cell viability was calculated with the equation [1 - (cell death/total) × 100]. For migration assays with niclosamide, colon cancer cells (5 × 10^4^) were seeded into transwell inserts (5.0 μm pore, BD Biosciences). After a 48 h culture period with or without niclosamide as indicated in the Figures, the upper side of the membrane was rubbed with cotton swab and the numbers of cells migrating to the basal side insert were stained with 0.25% crystal violet and counted. Cell counts were determined in five random fields.

### Reporter assay, immunoblot assay, immunoprecipitation

Colon cancer cells were transfected with 100 ng of reporter gene constructs and 1 ng of transfection control pRL-SV40-*Renilla*. Reporter activities were measured with a dual luciferase assay system (Promega) 48 h after transfection and normalized by measuring co-transfected *renilla* activity. Reporter gene activities are presented as light units relative to those obtained from negative control. For the western blot analyses, protein extracts were prepared in Triton X-100 lysis buffer. Antibodies against Snail (3895s, Cell Signaling, 1:2,000), GSK3β (ab82542, Abcam, 1:1,000), E-cadherin (#610181, BD Transduction, 1:5,000), β-catenin (#610154, BD Transduction, 1:5,000), APC (#2504, Cell Signaling, 1:1,000), LRP-6 (C5C7, Cell Signaling, 1:1,000), pLRP-6 (S1490, Cell Signaling, 1:1,000), and alpha-Tubulin (LF-PA0146A, Ab Frontier, 1:5,000) were obtained from the commercial vendors. For immunoprecipitation assay for Axin2, doxycycline-inducible His-tagged Axin2 expression vector were stably transfected into MCF-7 cells as described previously [8]. Whole cell Triton X-100 lysates were incubated with Ni-NTA beads (Invitrogen) with different doses of niclosamide. The recovered proteins were resolved by SDS-PAGE and subjected to immunoblot analysis for GSK3 and input (1/20 volume) control. The protein abundances of Snail and GSK3 were determined from nuclear-cytosolic fractionation of protein lysates with hypotonic buffer as described previously [4, 8]. Briefly, the colon cancer cells (1 × 10^6^) were collected into microcentrifuge tubes. The PBS-washed cells were treated with 400 μl of hypotonic buffer (10 mM HEPES, pH7.9; 10 mM KCl; 1 mM DTT with protease inhibitors) on ice for 5 min. The cell membrane was ruptured by adding 10% NP-40 to a final concentration of 0.6%, then vigorously vortexed for 10 sec followed by high-speed centrifuge for 30 sec. The supernatant cytosolic fractions were collected separately, and nuclear pellets were washed with ice-cold PBS twice. Nuclear protein was extracted with hypertonic buffer (20 mM HEPES, pH7.9; 0.4 M NaCl; 1mM DTT with protease inhibitors) for 15 min on ice followed by high-speed centrifuge.

### Cell-free Axin-FITC fluorescence (AFF) assay

His-tagged recombinant GSK3β was obtained from sf9 insect cells as described previously [6]. The FITC-conjugated 19-mer Axin peptide (Axin1, 383-401, VEPQKFAEELIHRLEAVQR), which is reported to bind GSK3 as an amphipathic α-helix, was chemically synthesized (Peptron)[24]. His-tagged recombinant GSK3 (300ng) was immobilized to Ni-NTA beads followed by phosphate buffed saline (PBS, pH 7.4) 3 times. The synthetic Axin peptide (10 ng) with different concentrations of niclosamide was subjected to the beads with His-tagged recombinant GSK-3 to examine competitive binding of Axin peptide for 2 h at 4 °C. After PBS washing 3 times, the Ni-NTA beads were subjected to quantitative fluorescent measurement at an excitation wavelength of 488 nm and an emission wavelength of 525 nm. The fluorescent intensities are presented as relative fluorescence intensity to that obtained from negative control from triplicate experiments.

### Surface plasmon resonance (SPR) Analysis

SPR was conducted using the ProteOn™ XPR36 Protein Interaction Array system (Bio-Rad Laboratories, Inc., CA, USA). Purified recombinant GSK3β was immobilized on the ProteOn GLH sensor chip. Niclosamide or 19-mer wild type Axin peptide or mutant peptide (VEPQKAAEEAIHRAEAVQR, mutation underlined) were diluted by phosphate-buffered saline with Tween 20 and 1% DMSO at different concentrations and then flowed over the chip at a rate of 100 μl/min. Data were analyzed with the ProteOn Manager Software 2.0 using the standard Langmuir models for fitting kinetic data. The rate of complex formation is represented by the association constant (k_a_, in the unit of M^−1^s^−1^) and the rate of complex decay is represented by the dissociation constant (k_d_, in the unit of s^−1^), as given by Equation 1:

$$\text{A}+\text{B}\overset{Ka}{\underset{Kd}{⇄}}\text{sd}$$ (1)

A high-affinity interaction is characterized by a low dissociation constant (K_D_), rapid recognition and binding of the interactants (rapid “on rate,” or high k_a_), and the stability of complex formation (slow “off rate,” or low k_d_) as shown in the equation, K_D_ = k_d_/k_a_.

### Molecular docking study

Molecular docking calculations were performed using the Maestro 10.4 molecular docking suite. The crystal structure of the human (pTyr216)-GSK3β bound with an Axin peptide was obtained from the RCSB Protein Data Bank (PDB ID: 3ZDI). All water molecules and metal ions were removed, and hydrogen atoms were added to the protein. To sample different ligand protonation states at physiological pH, the Epik module was used. All compounds were energy-minimized using LigPrep and then docked to receptor structures using the standard precision (SP) module of the Glide docking module within the Schrödinger Suite. Prior to Glide docking studies, a receptor grid box was generated at the centroid of the co-crystallized ligands. Post-minimization was used to optimize the geometry of the poses.

### Microarray data analysis of NCI-60 cancer cell panel and quantitative RT-PCR

Publicly available gene expression profiling data from 9 different cancer tissues of NCI-60 panel (GSE29288), using Agilent-014850 Whole Genome Microarray 4×44k G4112F, were downloaded from the Gene Expression Omnibus (http://www.ncbi.nlm.nih.gov/geo/). The data set consists of 132 samples (12 breast, 12 central nervous system, 18 colon, 18 leukemia, 14 melanoma, 22 non-small cell lung, 14 ovarian, 4 prostate, 18 renal cell cancer cells). The data were normalized with the voom transformation after trimmed mean of M-values (TMM) as implemented in the R Bioconductor “limma” and “edgeR” packages. For visualizing the Axin2 expression value of 3 probes (A-24_P298027, A_23_P159395, and A-23_P148015), heatmap2 as implemented in the R Bioconductor “gplots” package was used. Total RNA was isolated using TRIzol reagent (Invitrogen) following the manufacturer's protocol. The SuperScript III synthesis kit (Invitrogen) was used to generate cDNA. Real-time quantitative PCR (qPCR) analysis for Axin2 transcript was performed with an ABI-7300 instrument under standard conditions and SBGR mix (n = 3). The expression of ΔCt value from each sample was calculated by normalizing with GAPDH. The primer sequences were 5′-AAGGGCCAGGTCACCAAAC-3′ for Axin2 forward, 5′-CCCCCAACCCATCTTCGT-3′ for Axin2 reverse, 5′- ATGGGTGTGAACCATGAGAAG-3′ for GAPDH forward, and 5′- AGTTGTCATGGATGACCTTGG-3′ for GAPDH reverse.

### *In vivo* assay and APC-MIN mice experiment

All animal experiments were performed in accordance with the Institutional Animal Care and Use Committee of Yonsei University and approved by the Animal Care Committee of the Yonsei University College of Dentistry and National Cancer Center Research Institute. Female athymic nude mice (6 weeks old) were used for xenograft assays. HCT116 (5 × 10^6^) and SW480 (5 × 10^6^) cells were resuspended in 100 μl of PBS and injected into flank subcutaneous tissue. The mice were randomly assigned to two groups and treated 24 h after transplantation intraperitoneally daily with vehicle or niclosamide in vehicle. For *in vivo* intraperitoneal application, niclosamide was dissolved in 10% Cremophor EL (BASF) and 0.9% NaCl. After colon cancer cell inoculation, mice were monitored daily and weighed twice weekly, then measured with calipers when the tumors became visible. Tumor volume was calculated with the equation (LXW^2^)/2, where L is the longer dimension of the tumor and W is the shorter dimension. APC^Min^ mice were produced by mating wild type C57BL/6J female mice with APC^MIN+^ male mice (Jackson laboratory strain 002020). APC^Min^ progeny were identified by a polymerase chain reaction-based assay and randomly assigned to subgroups at 3 weeks of age. For intraperitoneal administration, vehicle or niclosamide in vehicle (50 mg/kg) were injected daily (6 days/week), and the mice were monitored daily and weighed twice weekly. After 14 weeks end-point, the mice were sacrificed and entire intestines were removed. The intestinal segments were opened longitudinally with scissors, rinsed in saline, and then spread out individually. Tissue were fixed with 10% buffered formalin 24 h and then washed twice with 70% ethanol. Intestinal segments were examined using stereomicroscope. The number of adenoma (small, < 1 mm; medium 1~3 mm; large > 3 mm) was counted from each mouse. For oral administration, the APC-MIN mice were fed niclosamide mixed with 15% sugar gel vehicle daily (6 days/week) for 14 weeks. The number and size of adenomas were counted under stereomicroscope.

### EMT in tumor xenografts

Nude mice were subcutaneously inoculated with SW480 cells (5 × 10^6^) cells. When the tumors reached an average of 500 mm^3^, mice were randomized into three groups and given intraperitoneal injections of a vehicle or niclosamide in vehicle (50 mg/kg, 200 mg/kg) for 3 days prior to sacrifice. Tissue lysate was isolated using Pro-prep protein extraction solution (#17081, Intron) from tumor cryosections. Protein abundance of Snail and E-cadherin in tumor samples was detected by immunoblot assay.

### Statistics

All statistical analysis of cell viability, AFF assay, cell migration, and reporter assay was performed with two-tailed Student's t-tests; data are expressed as means and s.d. The double asterisks denote P < 0.01, one asterisk denoting P < 0.05. Statistical significance of animal experiments was determined using the Mann-Whitney test; data are expressed as mean and s.e.m. for tumor volume. No statistical method was used to predetermine sample size.

## SUPPLEMENTARY MATERIALS FIGURES


