# Supplementary material for: Niclosamide is a potential therapeutic for familial adenomatosis polyposis by disrupting Axin-GSK3 interaction
Source: Oncotarget. 2017 Mar 16;8(19):31842–55. doi: 10.18632/oncotarget.16252 (PMC5458252; doi:10.18632/oncotarget.16252)
Supplement: Supplementary file 1 [file oncotarget-08-31842-s001.pdf]

Niclosamide is a potential therapeutic for familial adenomatosis polyposis by disrupting Axin-GSK3 interaction

SUPPLEMENTARY MATERIALS

SUPPLEMENTARY FIGURES

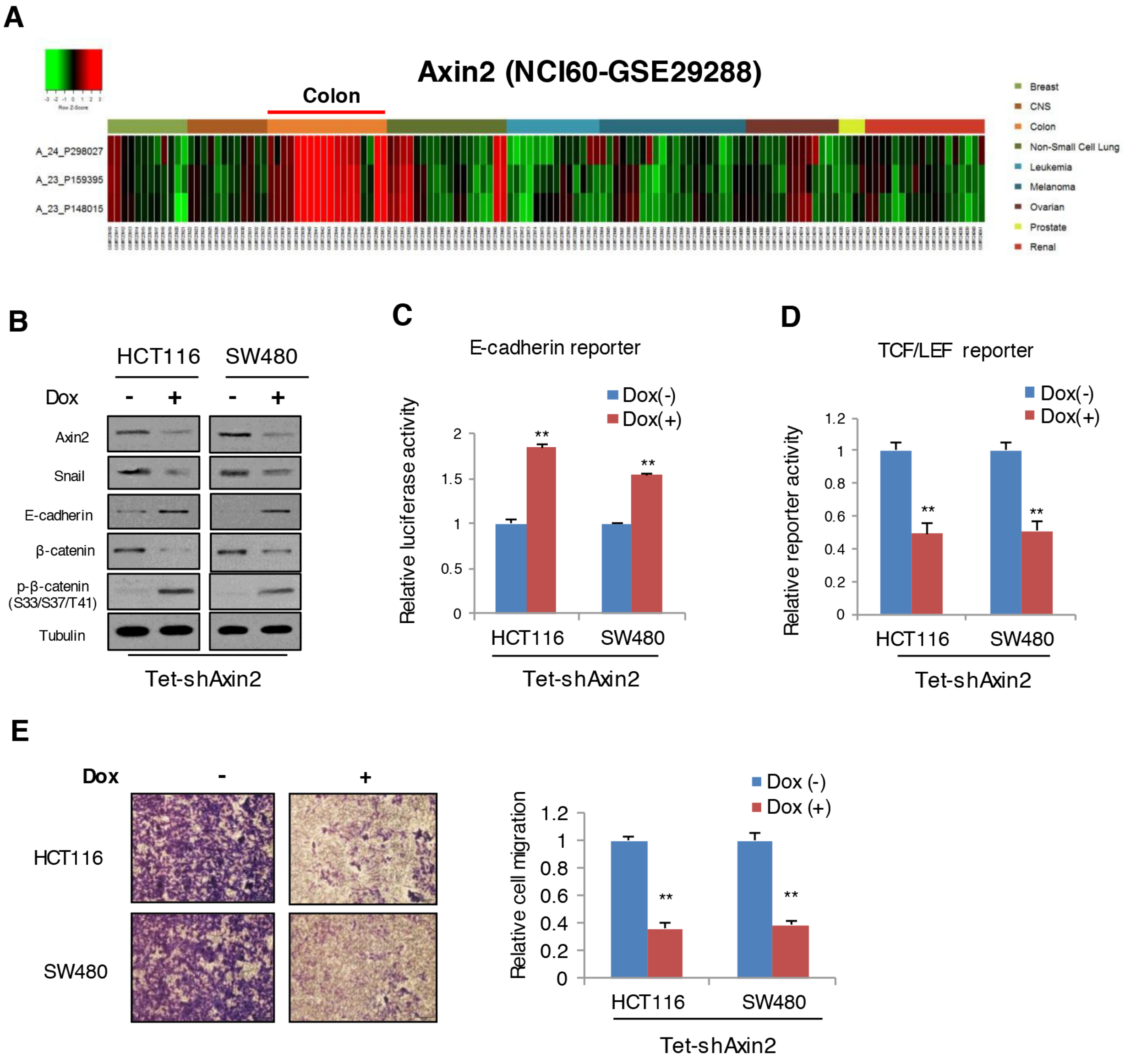

Supplementary Figure 1: Inducible knockdown of Axin2 reversed EMT and attenuated TCF/LEF transcriptional activity in colon cancer cells. **(A)** Axin2 transcripts abundance in human cancer cell lines. Publicly available dataset of NCI-60 cancer cell panel was analyzed to validate relative Axin2 transcripts level in colon cancer cells. Colon cancer cells were denoted as red bar. **(B)** The colon cancer cells were transduced with lentiviral Tet-inducible shRNA for Axin2, and immunoblot analysis was done of Axin2, Snail, and E-cadherin abundance in colon cancer cells. Reporter assay for E-cadherin proximal promoter having wild type E-box compared to 3xMut **(C)** and TCF/LEF Topflash **(D)** in colon cancer cells. **(E)** The migratory activity of colon cancer cells with inducible knockdown of Axin2 was determined by transwell migration assay (left panels), and the migration was quantified by crystal violet staining (right panel). Statistical significances compared to control are denoted as **, $P < 0.01$ by a two-tailed Student's t-test.

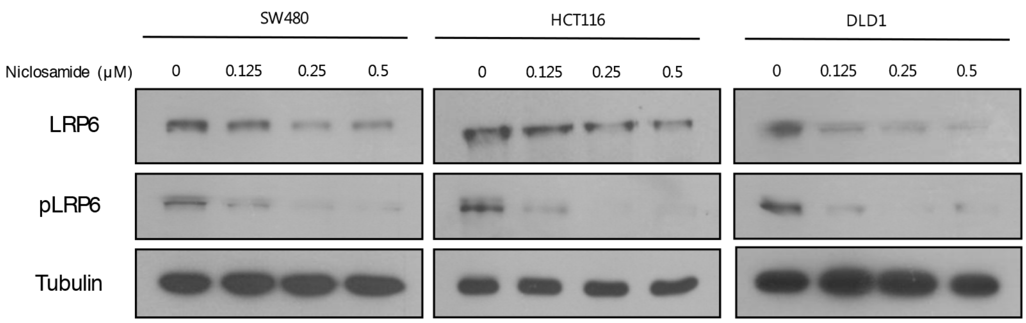

Supplementary Figure 2: Niclosamide suppressed phosphorylation of LRP6 in colon cancer cells. The colon cancer cells were treated with niclosamide at the indicated concentrations for 24 h, and protein abundances of LRP6 and phospho-LRP6 were determined by immunoblot assay.

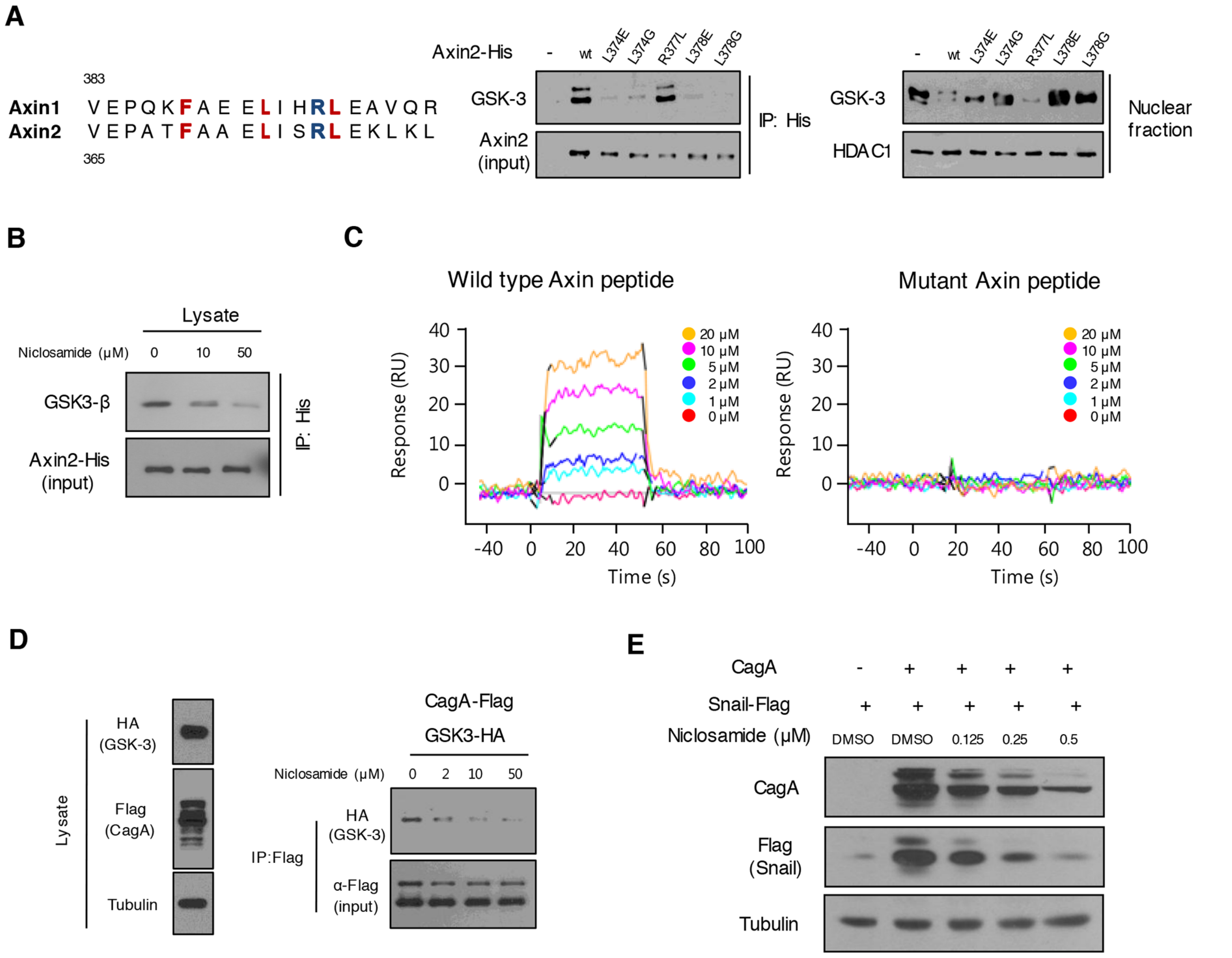

Supplementary Figure 3: Niclosamide binds to GSK3 and disrupts interaction with *H. pyroli* CagA. (A) Sequences alignment of GSK-3 binding domain on human Axin1 and Axin2 (left panel). Hydrophobic residues are denotes as red and putative hydrogen bond donor are denote as blue. His-tagged wild type and mutant Axin2 expression constructs (Leu for removal of hydrogen bond, Glu for hydrophilic substitute, and Gly for side chain size) were overexpressed in 293 cells, and subjected for immunoprecipitation to determine GSK-3 binding affinity (middle panel). The expression vectors were transfected into 293 cells, and endogenous nuclear GSK-3 abundance was measured by immunoblot analysis (rignt panel). **(B)** Cells were transfected with His-tagged Axin2, and the GSK3 binding activities in whole lysate were determined by Ni-Ti bead immunoprecipitation with increasing doses of niclosamide followed by immunoblot analysis for GSK3. **(C)** Binding interactions between the Axin peptide and GSK-3 measured by surface plasmon resonance (SPR). The SPR sensorgrams representing the concentration-dependent binding of the wild type (left panel) and mutant Axin peptide (right panel) with GSK-3 immobilized on a GLH chip. Five different concentrations were analyzed (0-20 μM). **(D)** The flag-tagged CagA expression vector and HA-tagged GSK-3β were co-transfected in 293 cells for a 48 h period (left panel). The GSK3 binding affinity of CagA with increasing doses of niclosamide was detected by immunoprecipitation with anti-Flag agarose following immunoblot analysis with anti-Flag (CagA input) and anti-HA (GSK-3β) (right panels). **(E)** The 293 cells were co-transfected with CagA and Snail expression vectors. The cells were treated with increasing doses of niclosamide and immunoblot analysis was done of CagA and Snail.

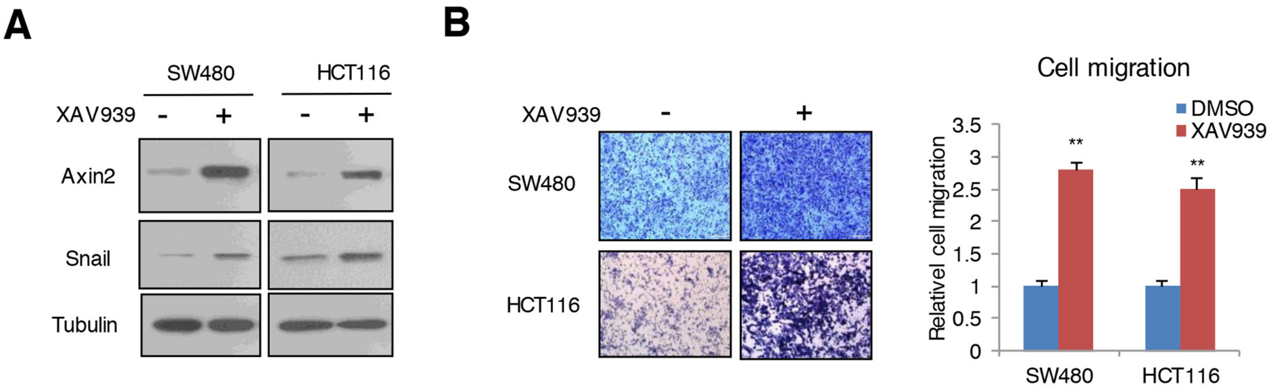

Supplementary Figure 4: Tankyrase inhibitor XAV939 stabilizes Axin2 and potentiates cell migration in colon cancer cells. **(A)** XAV939 (1 µM) increases the abundance of Axin2 and Snail in colon cancer cells. The cells were treated with XAV939 for 24 h and whole cell lysates were subjected to immunoblot analysis. **(B)** XAV939 increases migratory potential of colon cancer cells. The migratory activity of colon cancer cells treated with DMSO (-) or XAV939 (1 µM) was determined by transwell migration assay (left panels). Colon cancer cells (1×10^5) were pretreated with control (-) or XAV939 for 48 h, and cell migration was monitored for a 16 h period. The migratory cells were quantified with inverted microscope after crystal violet staining from 5 fields (right panel). Statistical significances compared to control are denoted as **, $P < 0.01$ by a two-tailed Student's t-test.

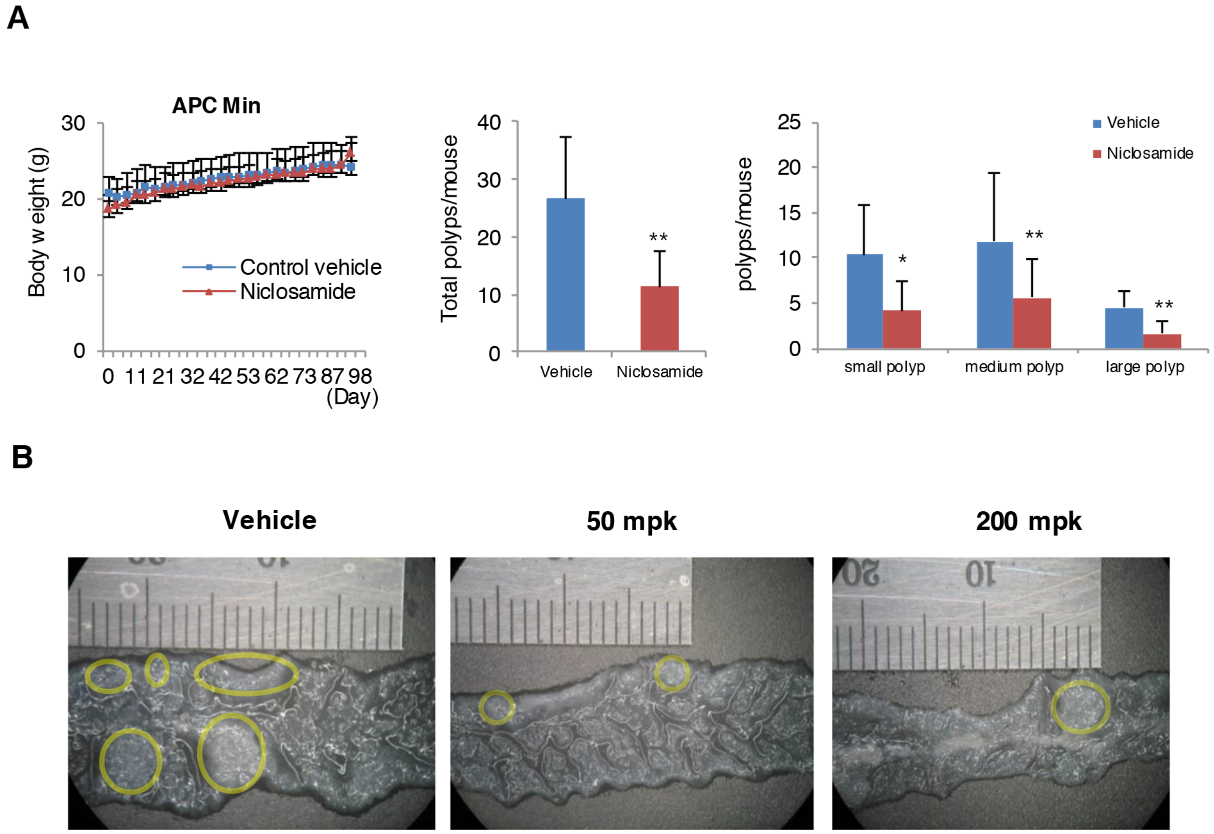

Supplementary Figure 5: Intestinal tumor suppression in APC-MIN mice by Niclosamide. **(A)** Effect of intraperitoneally administered niclosamide (50 mg/kg) on body weight (left panel), total tumor multiplicity (middle panel), and number of sized polyps (right panel). The APC-Min mice received Cremophor vehicle (n = 6) or 50 mg/kg niclosamide (n = 7) for 14 weeks end-point. Results are shown as means and s.d. One asterisk, $P < 0.01$; Two asterisks, $P < 0.001$ compared to the vehicle control by Mann-Whitney test. **(B)** Oral administration of niclosamide decreased adenoma formation in APC-Min mice. Low power stereomicroscopic images of representative intestines that showed the median adenoma value for each group. Circles indicate adenoma foci in mouse intestine.
